# An Exposition of the Signs and Symptoms of Pregnancy

**Published:** 1857-09

**Authors:** 


ART. IV.—*An Exposition of the Signs and Symptoms of Pregnancy:* With some other papers on subjects connected with Midwifery. By W. F. MONTGOMERY, A.M., M.D., M.R.I.A., Professor of Midwifery in the King and Queen's College of Physicians in Ireland, &c. &c. &c. From the Second London Edition. Pp. 568, Philadelphia: Blanchard & Lea. 1857.

THE work of the distinguished Irish Professor requires no commendation at our hands. As an exposition of the signs and symptoms of pregnancy, it has no rival in any language; and surely this is saying a great deal, considering the able treatises that have appeared upon the subject in France and Germany. The work indeed has become classical. The first edition, which consisted chiefly of a reprint of the admirable article which the author had furnished upon the signs and symptoms of pregnancy, to the Cyclopædia of Practical Medicine, was exhausted several years ago, and it was therefore with no ordinary satisfaction that we hailed the appearance of the present treatise, which may, in truth, be regarded as almost a new book. Much of the work has been entirely rewritten, the former edition has been revised, and a large amount of new matter has been added, thus increasing the size of the volume to double its original dimensions. Dr. Montgomery sought information wherever he could find it, at the bedside of a large private and hospital practice, and among distinguished medical men, both at home and abroad, in England, Scotland, France, Germany, Denmark, Sweden, Norway, and America. Under the head of the corpus luteum he frequently alludes, in appreciating terms, to the valuable labors of our countryman, Professor Dalton, of New York; whose researches have thrown so much light upon the nature and structure of that interesting formation. The work is written with great elegance, and abounds in the most valuable information upon every subject of which it treats. It is illustrated by a number of wood-cuts, and by two beautifully colored plates, representing the appearances of the corpus luteum in the various stages of its development.

The Philadelphia publishers deserve great credit for the manner in which they have brought out the American edition; they have spared no pains or expense to make it fully equal to the British. We are particularly gratified in not seeing any parasitical addenda, as they are called by Dr. Holmes, clinging to it: "no pediculus in the title-page—no pulex in the shape of a teasing footnote—no ascaris in the form of an appendix." The matter is wholly the author's, without any interpolation or distortion of any kind, pure and undefiled as it fell from his own hands. We hope the day is not distant when this great publishing house, and every other establishment of a similar character in the country, will see the propriety of issuing foreign works upon the strength of their own intrinsic merits, without the name of an American doctor, whose only merit too often consists in his bare-faced impudence in *indorsing*, as it has been modestly styled by a neighboring critic, the author of the book upon which he attempts to ride into a little temporary notoriety.

Want of space compels us to forego any further remarks upon Dr. Montgomery's work; at some future time we shall recur to it with a view of presenting a brief analysis of its valuable contents. Meanwhile we cordially recommend it to our readers as a production that should be in every Physician's library.

